# Impact of Voltage Application on Degradation of Biorefractory Pharmaceuticals in an Anaerobic–Aerobic Coupled Upflow Bioelectrochemical Reactor

**DOI:** 10.3390/ijerph192215364

**Published:** 2022-11-21

**Authors:** Qiongfang Zhang, Mei Wu, Nuerla Ailijiang, Anwar Mamat, Jiali Chang, Miao Pu, Chaoyue He

**Affiliations:** 1 Key Laboratory of Oasis Ecology of Education Ministry, College of Ecology and Environment, Xinjiang University, Urumqi 830017, China; 2 Xinjiang Jinghe Observation and Research Station of Temperate Desert Ecosystem, Ministry of Education, Urumqi 830017, China; 3 School of Chemical Engineering and Technology, Xinjiang University, Urumqi 830017, China; 4 Division of Environmental Engineering, School of Chemistry, Resources and Environment, Leshan Normal University, Leshan 614000, China

**Keywords:** bioelectrochemical reactor, pharmaceutical removal, applied voltages, electrode transition, bacterial community

## Abstract

Diclofenac, ibuprofen, and carbamazepine are frequently detected in the environment, where they pose a threat to organisms and ecosystems. We developed anaerobic–aerobic coupled upflow bioelectrochemical reactors (AO-UBERs) with different voltages, hydraulic retention times (HRTs), and types of electrode conversion, and evaluated the ability of the AO-UBERs to remove the three pharmaceuticals. This study showed that when a voltage of 0.6 V was applied, the removal rate of ibuprofen was slightly higher in the system with aerobic cathodic and anaerobic anodic chambers (60.2 ± 11.0%) with HRT of 48 h than in the control systems, and the removal efficiency reached stability faster. Diclofenac removal was 100% in the 1.2 V system with aerobic anodic and anaerobic cathodic chambers, which was greater than in the control system (65.5 ± 2.0%). The contribution of the aerobic cathodic–anodic chambers to the removal of ibuprofen and diclofenac was higher than that of the anaerobic cathodic–anodic chambers. Electrical stimulation barely facilitated the attenuation of carbamazepine. Furthermore, biodegradation-related species (*Methyloversatilis, SM1A02, Sporomusa*, and *Terrimicrobium*) were enriched in the AO-UBERs, enhancing pharmaceutical removal. The current study sheds fresh light on the interactions of bacterial populations with the removal of pharmaceuticals in a coupled system.

## 1. Introduction

Pharmaceutical and personal care products (PPCPs) are emerging pollutants, and their potential impact on the environment has attracted great attention. Non-steroidal anti-inflammatory drugs (NSAIDs) diclofenac and ibuprofen are among the most common pain relief medications in the world, accounting for 22% and 51% of total NSAIDs consumption, respectively [1]. Carbamazepine is used to treat epileptic seizures and depression. Due to the widespread use of these three drugs, they have become the most frequently detected PPCPs in the environment [2]. For example, diclofenac and ibuprofen were revealed to be present in trace concentrations ranging from 2 to 18,000 ng/L in river water [3]. The detection rate of carbamazepine in the Nansi Lake basin in China was 100% [4]. Wastewater treatment plants (WWTPs) are not effective in removing these three pharmaceuticals. Ashfaq et al. [5] studied the removal of various PPCPs at Xiamen WWTPs and found that the removal efficiency of diclofenac and carbamazepine was less than 50%. Due to the trace concentration (ng/L-μg/L) of PPCPs in environmental media (groundwater, surface water, etc.), the threats they pose to organisms or ecosystems are often neglected. A study suggested that diclofenac and ibuprofen impaired the cardiovascular development of zebrafish at low environmentally relevant concentrations between 0.04 and 25.0 μg/L [6]. Chaturvedi et al. [7] found that diclofenac, ibuprofen, and carbamazepine cause antibiotic resistance and the emergence of antibiotic-resistant genes. Additionally, research has suggested that carbamazepine and diclofenac containing amide and chloro groups are particularly persistent in the environment [8]. Hence, new and efficient technologies to remove ibuprofen, diclofenac, and carbamazepine from the environment are urgently needed.

In recent years, various methods have been applied to remove PPCPs from the environment, such as adsorption, advanced oxidation processes, membrane filtration, and many other technologies. However, there are presently evident disadvantages related to these technologies. For example, adsorption treatment is unable to ultimately degrade PPCPs, and adsorbent requires treatment to eliminate secondary pollution [9]. Advanced oxidation processes have attracted attention as they can completely mineralize or decompose most organic matter [10], but they are cost-prohibitive for full mineralization of PPCPs. Traditional biological methods are widely used because of their low operating costs and minimal environmental impacts [11], but they usually involve time-consuming techniques.

Bioelectrochemical systems (BESs) have shown excellent results for the degradation of recalcitrant compounds, and they are viewed as a promising way to remove PPCPs [11]. For example, Yang et al. [12] used biofilm electrode reactors to degrade sulfadiazine and found that 20 mg/L sulfadiazine eliminated rapidly within the first 3 h. Even high concentrations (160 mg/L) were rapidly removed after 24 h of system operation. Furthermore, the use of biocathodes also avoids the production of highly toxic byproducts from nitrofurazone [13]. Previous studies found that the mean total nitrogen removal rate reached the maximum of 0.65 kg/m^3^·d at the optimal voltage of 1.5 V, while higher voltage intensity (2.5 V) caused the inhibition of nutrient removal in the system. Under high voltage conditions, the relative abundance of some genera involved in pollutant removal such as *Planctomycetes* significantly reduced, while the relative abundance of the *Bacteroidetes* genus only showed a slight fluctuation [14]. Consequently, it is necessary to conduct a more comprehensive study on the changes of environmental operating conditions (e.g., intensity of electrical stimulation, microbial metabolic activity) when using BESs to treat pollutants. In addition, there have been no published data on the impact of electrode conversion on the elimination of PPCPs. Chen et al. [15] developed a novel reactor with built-in electrodes and discussed the advantages of internal electrode degradation of PPCPs, but did not report whether the lack of electrode conversion would have an impact on the removal of pharmaceuticals. Previous studies found that the application of BESs as an independent unit to treat wastewater did not achieve good performance [16]. Hence, Das, A. et al. [17] investigated combinations of BESs with the aerobic biological system to improve the degradation efficiency of azo dyes. In addition, Jiang et al. [18] found that a BES coupling system could effectively enhance the dechlorination of nitrobenzene, and its efficient and stable performance highlighted the potential of the BES coupling system, especially for the removal of refractory pollutants. However, there have been few comprehensive studies on the treatment of pharmaceutical wastewater with coupling reactor. Therefore, in this study, a novel anaerobic–aerobic coupled upflow bioelectrochemical reactor (AO-UBER) was developed to enhance the removal of ibuprofen, diclofenac, and carbamazepine from pharmaceutical wastewater. The main research aims were: (1) to determine the effect of different applied voltages and HRT on the removal of these pharmaceuticals, (2) to investigate the impact of electrode conversion on the elimination of pharmaceuticals, and (3) to analyze the responses of bacterial communities to AO-UBERs.

## 2. Materials and Methods

### 2.1. Reactor Configurations

**Figure 1:**
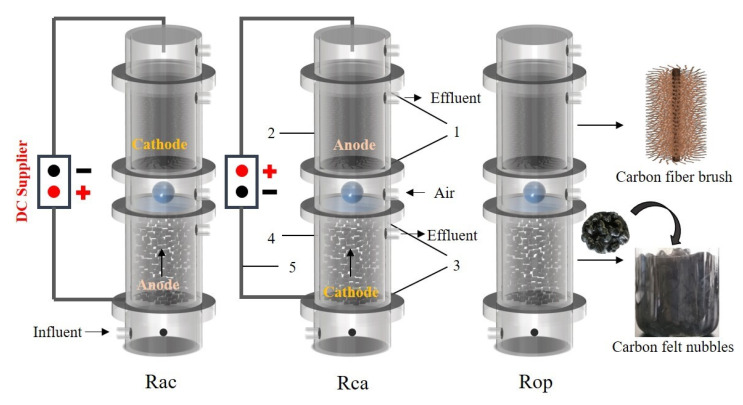
Schematic diagram of the experimental reactors (Rac: anaerobic anodic/aerobic cathodic chamber; Rca: anaerobic cathodic/aerobic anodic chamber; Rop: control reactor with open circuit). 1, titanium mesh (screen mesh size of 5 mm × 5 mm); 2, carbon fiber brush; 3, titanium mesh (screen mesh size of 2 mm × 2 mm); 4, carbon felt nubbles; 5, titanium plate.

Six AO-UBERs are depicted in Figure 1 and Figure S1. The reactors were constructed of plexiglass and had an effective capacity of 680 mL. the reactors’ specific configurations have been described previously [19]. Carbon fiber brush (CFB; aerobic biofilm carriers; 4.5 cm in diameter and 10 cm in length) and carbon felt nubbles (CFN; anaerobic biofilm carriers; 10 mm × 10 mm × 10 mm) were positioned in the aerobic and anaerobic zones, respectively, for bacterial growth. The total weight of each reactor with fillers was 580 ± 10 g.

### 2.2. Bacterial Inoculate and Reactors Operation

The aerobic and anaerobic sludge was collected from Hedong WWTP in Urumqi, Xinjiang, China. The sludge was first domesticated in our laboratory: aerobic sludge was placed in an open plastic bucket, aeration was continued within the bucket to keep the dissolved oxygen (DO) at about 4 mg/L. Aeration was stopped for 30 min daily to precipitate the aerobic sludge. Then the supernatant was discharged and equivalent distilled water added. This process lasted for 10 days. The anaerobic sludge was placed in a plastic bucket with a lid, the supernatant was discharged daily and equivalent distilled water was added, stirring evenly. This process lasted for 5 days. The following nutrients were used during domestication (pH 7.2 ± 0.3): 780 mg/L of CH_3_COONa, 47.72 mg/L of NH_4_Cl, 17.60 mg/L of K_2_HPO_4_ as sole carbon, nitrogen, and phosphorous sources, respectively, sustaining COD/N/P = 200/5/1. An additional 1 mL/L of trace element solution was introduced, which contained 100 mg/L of FeSO_4_·7H_2_O, 15 mg/L of CuSO_4_·5H_2_O, 161 mg/L of ZnSO_4_·7H_2_O, 8 mg/L of MnSO_4_·7H_2_O [20]. After the above process, the sludge supernatant was screened through filters of 200 mesh to remove impurities and then mixed with the above nutrients. The mixture was injected continuously from the bottom and middle of the reactors into the aerobic and anaerobic zones. The AO-UBERs were operated for 20 days during the process to ensure mature biofilm formation.

After sludge acclimation, the synthetic pharmaceutical wastewater was injected into the bottom of the reactors. The synthetic pharmaceutical wastewater consisted of carbamazepine (1 mg/L), ibuprofen (1 mg/L), and diclofenac (1 mg/L), nitrogen sources, phosphorus sources, and trace element solution as mentioned above. The concentration of the pharmaceuticals used was based on the detected concentration of the pharmaceuticals discharged from WWTPs into the water body, or slightly above the detected concentration [21]. All experiments were conducted at constant ambient temperature (20 ± 2 °C).

Intermittent electrical stimulation mode was used on four AO-UBERs (12 h ON/12 h OFF), based on our previous studies which demonstrated that intermittent electrical stimulation could be beneficial to maintain greater evenness for functional stability of microbial communities during the treatment processes of biorefractory organics, and most electroactive bacteria are enriched in the intermittent electrical stimulation mode [22,23]. The other two reactors were operated with an open circuit. Each operating condition had two identical reactors. There were three types of reactors: Rac, Rca, and Rop, which are shown in Figure 1. Rac was equipped with anaerobic anodic/aerobic cathodic chambers containing R1 and R2, while Rca was equipped with anaerobic cathodic/aerobic anodic chambers containing R3 and R4. Rop was operated with an open circuit containing R5 and R6.

Table 1 summarizes the operational conditions. In stage 1, the six reactors were fed synthetic pharmaceutical wastewater with 24 h of HRT and without voltage application. The system was initiated until the removal efficiencies were stabilized, and as there was a gap between the reactors, it was necessary to group them. The two reactors with the highest pharmaceutical removal efficiencies were used as control reactors with an open circuit Rop, and in stage 2, 0.3 V was applied on the other two kinds of reactors, Rac and Rca. In stage 3, the voltages applied to Rac and Rca were increased to 0.6 V. During the electrical stimulation process, the organic loads were adjusted from 24 h HRT to 48 h HRT, with the aim of judging the effect of electrical stimulation on drug removal in the short term. After the removal, efficiencies were stable under 0.6 V with 48 h HRT condition, and the voltage was sequentially added to 0.9 V and 1.2 V in stages 4 and 5. Previous research suggests 1.23 V is the minimum voltage for hydrogen evolution from electrolytic water and found that excessive voltage (1.2 V) will significantly inhibit the performance of the activated sludge system. In addition, based on our previous research results and the REDOX potentials of diclofenac (0.4–0.7 V) and ibuprofen (redox potential >100 mV) on carbon-felt biocathode, we chose the voltage gradient of 0.3 V, 0.6 V, 0.9 V, and 1.2 V [23,24,25].

### 2.3. Chemicals and Analytical Methods

Ibuprofen (C_13_H_18_O_2_, 98% purity), diclofenac (C_14_H_11_Cl_2_NO_2_, 98% purity), and carbamazepine (C_15_H_12_N_2_O, 98% purity) were bought from Shanghai Beijisi Biotechnology Center (Shanghai, China). The CFBs were purchased from the Metal Materials Company (Kunming, China) and CFNs were obtained from Beijing Jinglongte Carbon Technology Co., Ltd. (Beijing, China). All other compounds were bought from Urumqi Kehua Weiye Biotechnology Co., Ltd. (Urumqi, China).

A multimeter was employed to measure the electric current and voltage (UNI-T, UT39D, Yuride Technology Co., Ltd., Shanghai, China) and the pH value and DO concentration were measured using a meter (Leici, PHB-260, and JPB-607, Revitalize the flow instrument factory, Yuyao, China). The fluctuations in pH value and DO concentrations are shown in Figures S3 and S4. The COD concentrations were quantified by potassium dichromate titration [26]. Figure S5 showed the COD removal efficiencies during five stages, and the COD removal efficiencies of the three types of reactors remained stable. At regular time intervals, a liquid sample of 5 mL was taken from the anaerobic and aerobic zones of each reactor, filtered through 0.22-μm pore diameter syringe filters, and maintained at 4 °C until analysis. The detection and qualification of the three pharmaceuticals were performed using high-performance liquid chromatography (HPLC, Agilent) with an ultraviolet detector. An Agilent Eclipse XDB-C_18_ column (4.6 mm × 150 mm, 5 µm) was used. The mobile phase was 0.01% formic acid water and acetonitrile (45:55, *v*/*v*) with an injection volume of 20 μL at a flow rate of 0.6 mL/min. A detecting wavelength of 224 nm was used. The calibration curves of the three pharmaceuticals are shown in Figure S2.

### 2.4. Bacterial Community Analysis

Eight specimens, including the original inoculating seed sludge and biofilms at the last stage, were gathered in order to conduct analysis of the bacterial communities. S.An and S.Ae were assigned to the samples taken from the original anaerobic and aerobic sludge, Rac.Ae.C, Rca.Ae.A, and Rop.Ae gathered from the aerobic zones of Rac, Rca, and Rop, and Rac.An.A, Rca.An.C, and Rop gathered from the anaerobic zones of Rac, Rca, and Rop, respectively. The total number of bacterial communities was determined using real-time quantitative polymerase chain reaction (qPCR). Biofilms were collected from the cathodic and anodic chambers of the AO-UBERs and were used to quantify the total bacteria and 16S rRNA gene copy numbers per gram carbon-fiber brush or gram carbon-felt nubble to determine target bacterial abundances. For high-throughput sequencing, 16S rRNA genes were amplified using primers 515F (5′-GTGCCAG CMGCCGCGGTAA-3′) and 806R (5′-GGACTACHVGGGTWTCTAAT-3′) targeting the V3–V4 chamber of the 16S rRNA gene. The DNA extraction and qPCR procedures were identical to those published in our earlier study [19].

For the microbial analysis, the raw data were first quality filtered using Trimmomatic (version 0.33), then the primer sequences were identified and removed using Cutadapt (version 1.9.1), followed by splicing and removal of chimeras from double-ended reads, using USEARCH (version 10) (UCHIME, version 8.1), resulting in high-quality sequences for subsequent analysis. Sequences were clustered at the 97% similarity level using USEARCH (version 10.0), and by default, the operational taxonomic units (OTUs) were filtered at a threshold of 0.005% of the number of all sequences sequenced. This experiment included alpha diversity analysis, using the evenness index to measure the evenness of community species.

### 2.5. Statistical Analysis

The statistical analysis was implemented using SPSS (version 25.0, SPSS Inc., Chicago, IL, USA). One-way ANOVA and Tukey testing at a 0.05 probability level were applied to evaluate the significance of differences in pharmaceutical removal rates among the reactors.

## 3. Results

### 3.1. The Overall Elimination Efficiency of the Coupled System

**Figure 2:**
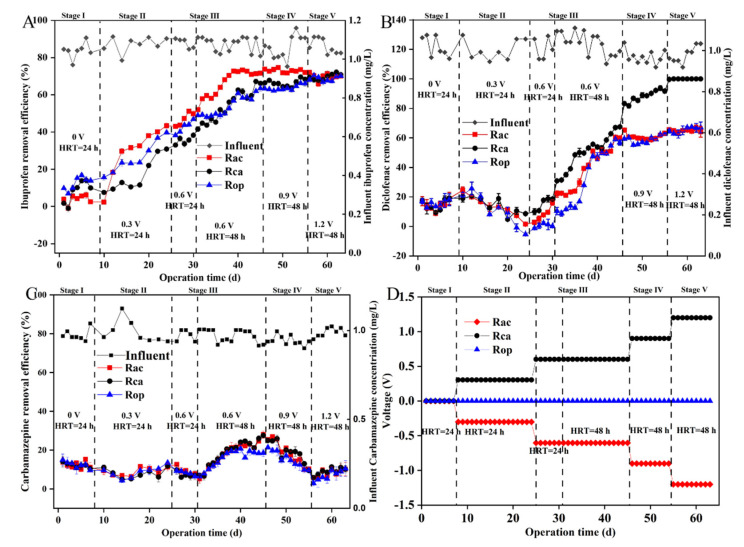
Ibuprofen (**A**), diclofenac (**B**), and carbamazepine (**C**) removal performances of AO−UBERs and the applied voltages of Rac, Rca, and Rop (**D**), with the influent pharmaceutical concentration in right y axis.

The total removal efficiency of ibuprofen, diclofenac, and carbamazepine in the AO-UBERs and the influent pharmaceutical concentrations are presented in Figure 2. It was noted that the three types of reactors had different pharmaceutical removal performances due to the HRT and voltages applied. In stage 1, the HRT was 24 h, all reactors were operated with an open circuit. The efficiencies of diclofenac and carbamazepine removal by the three groups of reactors were kept at a similar level of approximately 14.9 ± 3.5% and 12.7 ± 1.8% (*p* > 0.05) when the diclofenac and carbamazepine concentrations were 1.02 ± 0.08 mg/L and 1.05 ± 0.05 mg/L, respectively. However, ibuprofen (1.08 ± 0.16 mg/L) removal efficiencies among reactors widely ranged from 3.8 ± 2.5% to 12.5 ± 3.4% (*p* < 0.05), and it was clear that the three kinds of reactors performed differently in terms of ibuprofen elimination. To verify whether electrical stimulation could help with the removal of recalcitrant pollutants, four reactors with low removal efficiencies were chosen for voltage appication.

In stage 2, 0.3 V was applied and the HRT remained 24 h. It was observed that ibuprofen removal in all reactors slightly increased in Rac (from 2.38 ± 0.13% to 32.6 ± 0.6%), Rca (from 7.58 ± 0.30% to 11.6 ± 0.7%), and Rop (from 15.6 ± 1.1% to 23.6 ± 0.6%) over 10 days. However, the removal of diclofenac in all reactors decreased from 21.6 ± 2.3% to 3.9 ± 4.7%. In addition, carbamazepine removal efficiency in all reactors improved from 10.0 ± 0.7% to 12.2 ± 0.9% over time.

In stage 3, the voltage was increased to 0.6 V and the HRT was 24 h for the first ten days. The removal efficiencies of ibuprofen, diclofenac, and carbamazepine were 46.3 ± 3.2%, 6.2 ± 2.6%, and 9.6 ± 1.9% in Rac; 34.7 ± 1.4%, 14.4 ± 3.9%, and 7.4 ± 1.4% in Rca; and 41.5 ± 2.4%, 0.7 ± 1.3%, and 8.7 ± 0.5% in Rop, respectively, over five days. It was observed that when the HRT was 24 h, although the elimination of ibuprofen was enhanced, promotion of diclofenac and carbamazepine removal efficiencies was not apparent. To maximize the target contaminant removal efficiency, the HRT was increased from 24 h to 48 h. The ibuprofen removal efficiencies increased to 60.2 ± 11.0% (Rac), 51.4 ± 11.3% (Rca), and 51.2 ± 8.7% (Rop). After the increase of HRT, removal of diclofenac was enhanced in Rca (49.8 ± 0.5%) compared with Rac (39.3 ± 1.3%) and Rop (27.9 ± 0.4%). The highest removal efficiency was achieved by Rca. The removal of carbamazepine gradually increased from 7.9 ± 0.4% to 28.1 ± 1.9% in Rac, 7.1 ± 2.3% to 27.6 ± 0.04% in Rca, and 6.4 ± 0.5% to 18.6 ± 1.8% in Rop, and it was observed that the longer HRT was more beneficial for the removal of carbamazepine.

In stage 4, the voltage was increased to 0.9 V, and the HRT was 48 h. The removal efficiencies of ibuprofen were 72.9 ± 0.9% (Rac), 66.7 ± 1.9% (Rca), and 64.2 ± 1.8% (Rop). During this period, the removal efficiencies of diclofenac in Rac increased from 83.3 ± 1.3% to 99.7 ± 0.4%, while the removal efficiencies in Rca and Rop remained stable at 61.3 ± 2.3% and 59.3 ± 2.9%. It was shown that the removal efficiency of diclofenac in Rca was about 90%. However, the removal efficiencies of carbamazepine decreased from 25.5 ± 0.2% to 5.8 ± 0.4% in Rac, 24.9 ± 0.04% to 21.4 ± 0.7% in Rca, and from 21.4 ± 0.7% to 2.9 ± 1.3% in Rop.

In stage 5, the voltage increased to 1.2 V. For ibuprofen, the removal efficiency stabilized at 69.4 ± 1.6% (Rac), 69.7 ± 1.6% (Rca), and 68.8 ± 1.3% (Rop). Additionally, it was observed that the complete removal of diclofenac was achieved by Rca while the other removal efficiencies were 64.9 ± 0.9% (Rac) and 65.5 ± 1.9% (Rop). It was apparent that 1.2 V was the most favorable for the attenuation of diclofenac. For carbamazepine, the removal efficiencies were gradually increased from 6.4 ± 0.08% to 10.2 ± 3.5% in Rac, 7.48 ± 1.4% to 10.2 ± 1.8% in Rca, and 4.9 ± 3.9% to 10.6 ± 4.0% in Rop.

### 3.2. Pharmaceutical Removal in Cathodic and Anodic Chambers

**Figure 3:**
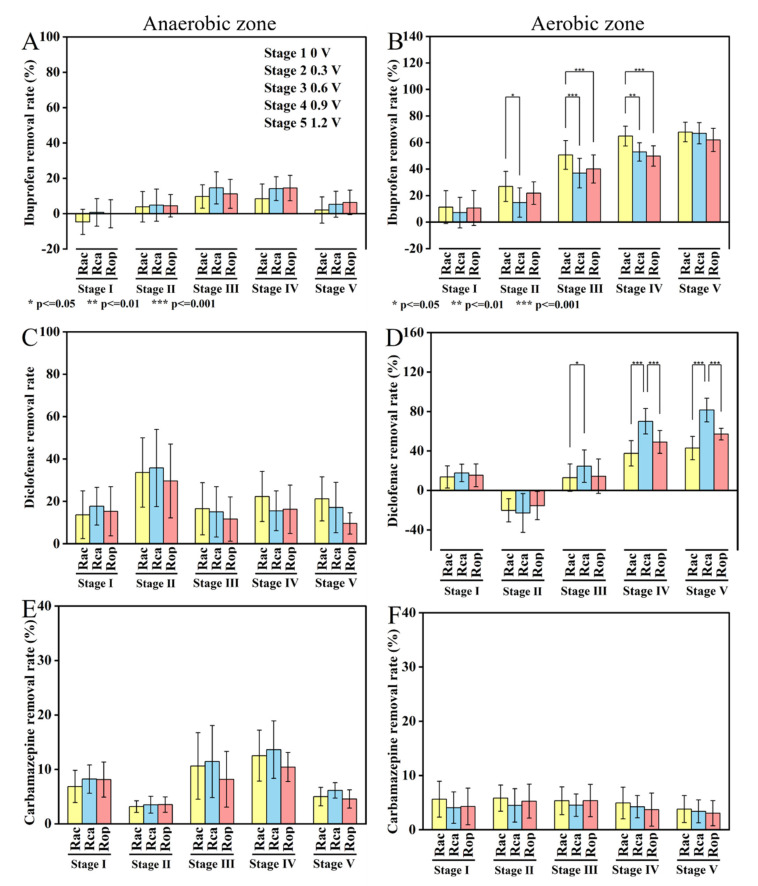
Ibuprofen (**A**,**B**), diclofenac (**C**,**D**), and carbamazepine (**E**,**F**) removal performances in anaerobic and aerobic zones of AO−UBER chambers.

Figure 3 indicates the removal performance for the three targets contaminant in the aerobic and anaerobic chambers of the AO-UBERs at different voltages.

As shown in Figure 3A,B, when all reactors were operated with an open circuit, the average removal efficiency of ibuprofen was 9.70% in the aerobic zones, with negative removal values of ibuprofen in the anaerobic zones. When the voltage was increased to 0.3 V, the contribution of the aerobic cathodic/anodic chambers (26.85%, 14.67%) to the removal of ibuprofen was higher than that of the anaerobic cathodic/anodic chambers (4.83%, 3.89%).The removal efficiency of ibuprofen by the control system was 21.85% in aerobic zones, lower than that of the aerobic cathodic chambers. When the voltage was increased from 0.6 V to 1.2 V, the removal efficiencies of ibuprofen decreased from 14.58%, 9.71% to 5.30%, 1.54% in anaerobic cathodic/anodic chambers. The removal efficiencies of ibuprofen increased from 50.59%, 36.91% to 67.87%, 66.95% in the aerobic cathodic/anodic chambers, higher than in the control system (40.04–61.97%) (*p* < 0.05).

As shown in Figure 3C,D, the average removal efficiencies of diclofenac were 16.60% and 18.65% in the aerobic zones and anaerobic zones, respectively, without applied voltage. When the voltage was increased to 0.3 V, the removal efficiencies of diclofenac were increased to 35.73% and 33.61% in the anaerobic cathodic/anodic chambers, which were higher than that in the control system (29.62%). However, three types of reactors showed negative removal efficiencies in the aerobic zones. When the voltage was increased to 0.6 V, it was observed that the aerobic cathodic/anodic chambers (33.18%, 47.48%) exhibited greater performance in diclofenac removal than the control system (29.76%). The removal efficiencies of diclofenac decreased to 15.04%, 16.50%, and 11.62% in the anaerobic cathodic/anodic chambers and the control system, respectively. When the voltage was increased from 0.9 V to 1.2 V, exceptional performance in terms of diclofenac removal was achieved in the aerobic anodic chambers (70.07–81.44%), higher than that in the control system (49.01–57.08%) (*p* < 0.05). In addition, the removal efficiencies of diclofenac were increased from 15.51% to 17.08% in the anaerobic cathodic chambers. The diclofenac removal efficiency in the anaerobic anodic chambers decreased to about 21.18%, remaining 11.64% higher than that in the control system.

As shown in Figure 3E,F, when all reactors were operated with an open circuit, the average removal efficiencies of carbamazepine were 4.66% and 7.73% in the aerobic zones and anaerobic zones, respectively. When the voltage was increased from 0.3 V to 1.2 V, the removal efficiencies of carbamazepine remained at a similar level about 4.57% in the aerobic cathodic/anodic chambers. The control system was 4.35% in the aerobic zones. Carbamazepine removal increased slightly when the voltage was enhanced from 0.3 to 0.9 V in the anaerobic cathodic chambers (3.16–12.52%) and anaerobic anodic chambers (3.49–13.62%), and was higher than that in the control system (3.15–10.43%). When the voltage was increased to 1.2 V, the removal efficiencies of carbamazepine were decreased to 5.00%, 6.12% and 4.56% in the anaerobic cathodic/anodic chambers and the control system.

### 3.3. Effect of Intermittent Supply on Bacterial Communities

#### 3.3.1. Total Bacteria Quantity

Six biofilm samples were obtained from the CFBs and CFNs after 64 days of operation, to measure the total bacteria in Rac, Rca, and Rop. The log_10_ (copies/g CFN or g CFB) of the six biofilm samples were 10.4, 10.3, 10.2, 10.1, 10.2, and 10.2 for Rac.Ae.C, Rca.Ae.A, Rop.Ae.O, Rac.An.A, Rca.An.C and Rop.An.O, respectively (Table 2).

#### 3.3.2. Analysis of Bacterial Diversity

As shown in the Table S1, the coverage values were all above 0.999, demonstrating that the bacterial diversity sequencing data in this experiment were highly reliable. As shown in Table 3, the two indices of anaerobic (295–340, 0.86–0.89) and aerobic (274–289, 0.92–0.94) biofilm samples were higher than the original anaerobic (235, 0.70) and aerobic sludge (271, 0.66). In addition, the evenness indices of anaerobic (0.89–0.97) and aerobic biofilm (0.94–0.95) in the applied voltage groups were higher than those of the anaerobic (0.86) and aerobic zones (0.92) in the control groups. However, it was shown that the OTU was between 312–340 (anaerobic) and 274–289 (aerobic) in the applied voltage biofilm samples, while values for the anaerobic and aerobic zones of the control groups were 295 and 275.

#### 3.3.3. Bacterial Community Structure Analysis

**Figure 4:**
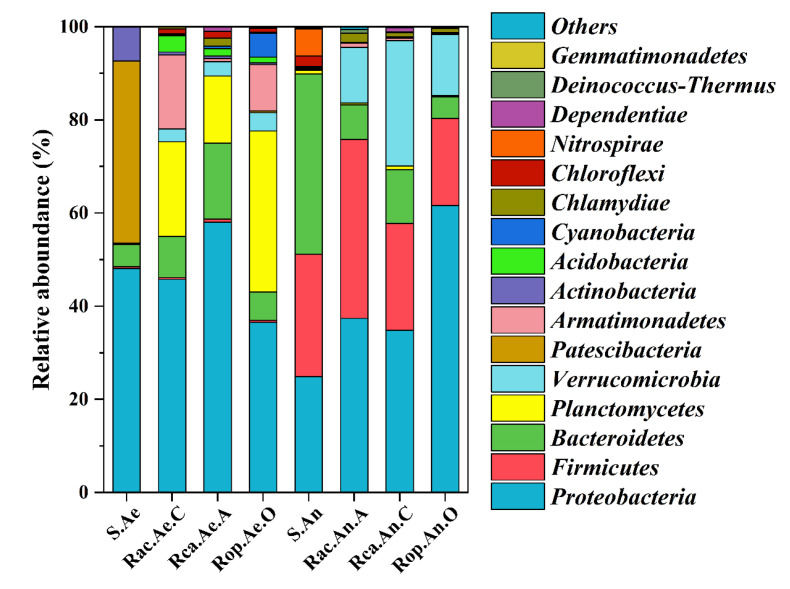
Relative abundances of bacterial community composition in eight samples at the phylum level, for aerobic and anaerobic zones. S.Ae and S.An were collected from initial aerobic and anaerobic sludge; Rac.Ae.C, Rca.Ae.A, and Rop.Ae.O were collected from the aerobic zones of Rac, Rca, and Rop; Rac.An.A, Rca.An.C, and Rop.An.O were collected from the anaerobic zones of Rac, Rca, and Rop, respectively.

The bacterial community structure of biofilms in Rac, Rca, and Rop, as well as the original sludge, are shown in Figure 4 at the phylum level. *Proteobacteria*, *Bacteroidetes*, *Planctomycetes*, *Armatimonadetes, Firmicutes*, and *Verrucomicrobia* were found to be the most common bacteria, although different samples had different dominant types.

At the phylum level, it was observed that *Proteobacteria* (24.9–61.6%), *Firmicutes* (18.6–38.4%) and *Bacteroidetes* (4.71–38.7%) were dominant in all anaerobic samples. In terms of anaerobic biofilms, it was found that the main bacteria were *Proteobacteria, Firmicutes, Bacteroidetes,* and *Verrucomicrobia*. *Firmicutes* and *Bacteroidetes* in Rac.An.A (38.40%, 7.41%) and Rca.An.C (22.9%, 11.6%) were in higher relative abundance than those in Rop.An.O (18.6%, 4.71%). However, *Proteobacteria* (61.6%) were dominant in the biofilm of Rop.An.O, whereas *Verrucomicrobia* (26.9%) dominated Rca.An.C.

Regarding the aerobic zones, *Bacteroidetes* (4.78%) and *Planctomycetes* (0.21%) were dominant in the original aerobic sludge, increasing from 6.08–16.31% and 14.8–34.5% in the aerobic biofilms. Additionally, the relative abundances of *Proteobacteria* and *Bacteroidetes* in Rca.Ae.A (58.0%, 16.3%) were higher than in Rac.Ae.C and Rop.Ae.O, while quantities of *Armatimonadetes* (15.9%, 9.95%) in Rac.Ae.C and Rop.Ae.O were higher than in Rca.Ae.A.

**Figure 5:**
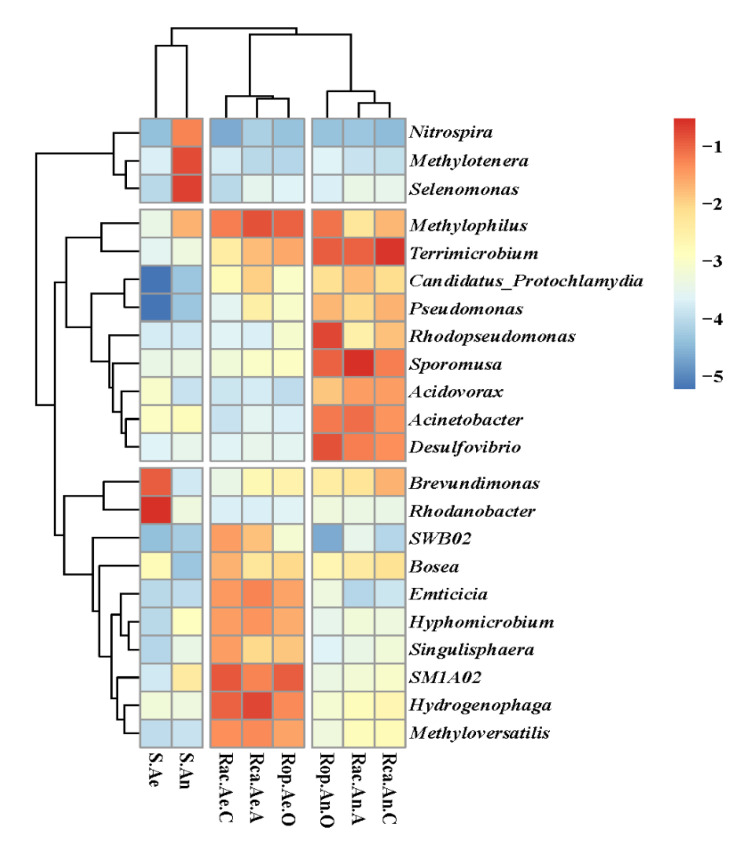
Heatmaps of bacterial communities of the eight samples at the genus level. S.Ae and S.An were collected from initial aerobic and anaerobic sludge; Rac.Ae.C, Rca.Ae.A, and Rop.Ae.O, were collected from the aerobic zones of Rac, Rca and Rop; Rac.An.A, Rca2.An.C, and Rop.An.O were collected from the anaerobic zones of Rac, Rca, and Rop.

The bacterial community structures were studied at the genus level to better understand the differences in particular microorganisms in the reactors and the original sludge. The dominating bacteria in the aerobic biofilm specimens were *Hydrogenophaga*, *Methylophilus*, *SM1A02*, *Methyloversatilis*, *SWB02*, *Emticicia*, *Hyphomicrobium*, and *Singulisphaera* with relative abundances of 5.51–19.3%, 6.92–15.7%, 5.88–12.5%, 3.14–4.74%, and 0.10–14.3%, 3.06–5.99%, 2.62–4.25%, and 1.07–3.66%, respectively (Figure 5). These abundances were higher than those in the original aerobic sludge (0.080%, 0.050%, 0.022%, 0.015%, 0.006%, 0.013%, 0.012%, and 0.012%, respectively). Meanwhile, *Sporomusa* (11.8–26.1%), *Terrimicrobium* (6.73–30.2%), *Desulfovibrio* (4.99–15.2%), *Acinetobacter* (4.27–8.70%), *Acidovorax* (1.50–3.73%), and *Rhodopseudomonas* (0.36–19.0%) predominated in anaerobic biofilm samples, in quantities that were greater than in the original anaerobic sludge samples (0.058%, 0.065%, 0.043%, 0.201%, 0.018% and 0.021%, respectively).

In terms of the anodic and cathodic chambers, it was found that the relative abundance of *Hydrogenophaga* (11.0–19.3%), *SWB02* (1.78–3.44%), *Methyloversatilis* (4.74–5.34%), and *Emticicia* (3.95–5.99%) were higher in the aerobic biofilm samples with applied voltage than in the Rop.Ae.O biofilm samples (5.51%, 0.10%, 3.14%, and 3.06%, respectively).

Moreover, the abundance of *Acidovorax* in Rac.An.A (3.72%) and Rca.An.C (3.73%) was higher than in Rop.An.O (1.50%). Interestingly, *Sporomusa* was found in abundance in the biofilm of Rac.An.A (30.2%), compared with anaerobic biofilms (6.73–11.0%). It was found that the *Terrimicrobium* was enriched in Rca.An.C (26.1%) higher than in the other reactors (11.1–13.0%).

## 4. Discussion

### 4.1. Operational Performance of the AO-UBERs

Rac significantly enhanced ibuprofen removal efficiency by about fifteen times (from 2.38 ± 0.13% to 32.6 ± 0.6%) at 0.3 V voltage, and the contribution of the aerobic cathodic chamber (26.85%) to the ibuprofen removal was higher in the control system (21.85%), which may be attributed to electrical stimulation enhancing the metabolic activity of aerobic microbes on the cathode. This is also supported by previous research [27] reporting that the •OH radical generated by aerobic cathodic electrolysis of H_2_O is the major oxidizing agent for the degradation of ibuprofen. A similar result was also obtained by Chang et al. who noted that the ibuprofen was broken by direct or indirect oxidation to form a series of small organic molecules. Then, these small organic molecules and intermediate products were completely decomposed into CO_2_ and H_2_O by microorganisms [28]. When the voltage was increased to 0.6 V, there were no significant differences observed in ibuprofen removal among the three types of reactors, and the average removal efficiency of ibuprofen was about 40.83%. This effect could be related to the exposure time of ibuprofen in the biofilm. This result was also similar to the literature [28] reporting that the effect of electrical stimulation intensity was obvious after HRT extension, and the removal efficiency of ibuprofen increased by 6.10% when HRT increased from 3 h to 3.5 h in an electric biological integration reactor. In addition, ibuprofen has a long half-life and inevitably needs more time to react with the biofilm [29]. When the HRT was increased from 24 h to 48 h, Rac could not only significantly increase the removal efficiency of ibuprofen to 60.2 ± 11.0% from 3.8 ± 2.5% at 0.6 V voltage, but also enable the removal efficiency of ibuprofen to reach stability more rapidly than Rop. Ibuprofen removal reached stabilization after 40 days in Rac, less than in Rop (55 days), and the aerobic cathodic chambers were the main contributors. A possible explanation of this phenomenon may be the presence of dissolved oxygen in the aerobic cathodic chambers. The indirect oxidation of ibuprofen by bacteria in the anaerobic anodic chambers transferred the electrons and ions to the aerobic cathode. The use of oxygen at the aerobic cathode supplied an additional electron acceptor for the bacterial metabolism [30], which accelerated the formation of electroactive biofilms and thus shortened the time required for bacterial acclimatization. In a previous study, it was found that using a single biodegradable system (a moving bed biofilm reactor), the removal rate of ibuprofen was only 14.33–31.33% [29]. Zhu et al. [31] used the sulfur/iron-mediated autotrophic denitrification system to degrade ibuprofen, and the ibuprofen removal rate was only 60.60% on the 46th day of the experimental period. The effluent concentration of ibuprofen decreased significantly (*p* < 0.05) with the operation of the system. After the 65th day, the removal rate of ibuprofen reached a relatively stable level. By comparison, the AO-UBERs used in this study could stabilize the ibuprofen removal rate faster and maintain a high level. Rop exhibited a stable ibuprofen removal efficiency of about 69.3% at 1.2 V voltage, similar to Rac and Rca, which could be the result of the long-term acclimation of the aerobic biofilm. Previous studies have reported that ibuprofen contains powerful electron donors (-OH), which can be removed effectively in aerobic conditions [32].

For diclofenac, the removal efficiencies in the three types of reactors decreased when the voltage was increased to 0.3 V. This could be attributed to the electrosorption at the electrode with opposite charge [33], which led to diclofenac being released back when it was adsorbed and saturated by the electrode. When the voltage was increased to 0.6 V, the removal efficiencies of diclofenac in Rac (39.3%) and Rca (49.8%) were higher than that in Rop (27.9%), but still not ideal, meaning that weak electric stimulation could not promote the removal of diclofenac by microorganisms. Moreover, the optimal removal voltages could be different for the different contaminants, as demonstrated in previous studies [22,34]. When the voltage was increased to 0.9 V, the removal efficiency of Rca was significantly improved, 32.2% higher than that of Rop. After increasing the voltage, more electrons were available to the attached microbes on the aerobic anodic, so diclofenac could be oxidized via direct electron transfer. Thus, the diclofenac removal rate increased [35]. When the applied voltage was 1.2 V, the complete removal of diclofenac in Rca was achieved, which was approximately 24.36% higher than that in Rop. This demonstrated that the optimum voltage for removing diclofenac in AO-UBERs was 1.2 V. The contribution of the aerobic cathodic/anodic chambers (42.87%, 81.44%) to the removal of diclofenac was higher than that of the anaerobic cathodic/anodic chambers (17.08%, 21.18%). Diclofenac was not easily degraded by oxidation, due to its special molecular structure containing two chlorinated strong electron-withdrawing groups. Dechlorination and further mineralization are two key steps in diclofenac degradation; dechlorination can reduce the stability of the chemical structure of diclofenac, and oxidation could easily transform the reductive intermediates into small organic molecules [36]. The complete degradation of diclofenac achieved by the use of AO-UBERs in this study may be due to the fact that the two chlorinated strong electron-withdrawing groups are more inclined to efficient oxidative removal in the aerobic anode. The anaerobic cathodic can serve as an electron donor to promote the microbial reductive dechlorination of diclofenac, while the aerobic anodic can be utilized as an electron acceptor to enhance its microbial oxidative degradation [37]. In our previous study, conductive carrier-supported biofilm was shown to strengthen the metabolic activity of aerobic microbes in the biofilm on the anode, and functional microbes were enriched under intermittent electrical stimulation [22], which is perhaps another reason for this situation. Qiu et al. [37] reported diclofenac degradation efficiency of 75.6% achieved using a Ru/Fe modified anode in a microbial fuel cell. Elsewhere, it was reported that the mineralization rate of diclofenac was only 69.6% in a Ru/Fe biocathode dual-chamber BES [36]. In contrast, the coupled system with aerobic anodic and anaerobic cathodic chambers used in our experiments could completely remove diclofenac at 1.2 V voltage.

In our study, it was observed that the average removal efficiencies of carbamazepine exhibited no significant difference between the applied voltage reactors and the control system, and the average removal efficiencies of carbamazepine were lower than 15% in the three types of reactors, indicating little effect of electrical stimulation on carbamazepine removal. Previous studies have demonstrated that due to the hydrophobicity of carbamazepine, the adsorption method is more favorable for its removal [38]. The average removal efficiency of carbamazepine for the three types of reactors was 7.79% in anaerobic zones, which was about 3.19% higher compared with that in aerobic zones. It has been reported that carbamazepine is more recalcitrant in aerobic conditions due to the electron-withdrawing groups of amide (–CONH_2_) [39].

### 4.2. Effect of Electrical Stimulation on Microbial Communities

It was speculated that the bacterial biomass was not influenced after the long-term operation of the electrically stimulated reactors compared with the open circuit reactors.

The OTU and evenness indices were calculated to describe the diversity and evenness of microbial communities. It can be seen that the more species in the community, the more uniform the distribution of various individuals and the higher the OTU index, indicating better diversity of the community [40]. The results above indicated that the bacterial species in the aerobic and anaerobic zones increased after long-term operation, which may be due to the enhancement of the metabolism of bacterial communities related to pharmaceutical removal, which is comparable to prior research [31]. This showed that except for the OTU index of the aerobic cathodic chamber in Rac being lower than that of the aerobic zone of Rop, the bacterial abundance and biodiversity both increased with electrical stimulation in AO-UBERs, suggesting that the anodic and cathodic chambers were capable of preserving the integrity of the bacterial population. It has also been reported that high community diversity of the bioelectrochemical system was capable of resisting the addition of toxic pollutants [41], thus realizing the removal of pharmaceuticals. Our study demonstrated that applied voltage could stabilize the bacterial community and resist toxicity when treating pharmaceuticals.

Through analyzing the structure of bacterial communities, it was shown that *Proteobacteria*, *Bacteroidetes*, *Planctomycetes*, *Armatimonadetes, Firmicutes*, and *Verrucomicrobia* were the most common bacteria, and all of these could also be discovered when removing pharmaceuticals from wastewater, according to earlier research [42,43,44]. This may be attributed to the fact that electrical stimulation is beneficial for the enrichment of different anaerobic and aerobic microorganisms. Similarly, it was found that *Proteobacteria* dominated aerobic biofilms after the addition of pharmaceuticals [45]. In addition, according to the findings, electrical stimulation significantly enriched particular microorganisms in the aerobic anodic and cathodic chambers of AO-UBERs. These results revealed that the functional bacteria in biofilms engaged in pharmaceutical removal were enriched over time.

Furthermore, the dynamic shifts in the species composition of the microbial communities and their main functions are revealed in Figure 5 and Table 4.

It was shown that electrical stimulation had a distinct influence on the removal of pharmaceuticals. The abundance of *Hydrogenophaga*, *Methyloversatilis,* and *Emticicia* in the biofilm of Rca.Ae.A was higher than in Rac.Ae.C and Rop.Ae, which may be attributed to the aerobic anodic chamber promoting the enrichment of functional microbes. For example, *Hydrogenophaga* could enhance dehydrogenase activity and the secretion of EPS, thereby resisting the toxicity of pharmaceuticals. In addition, it has often been found that electrical stimulation could enhance the use of hydrogen as an electron [46]. *Methyloversatilis* species are typical microbes that are essential for the oxidation of aromatic hydrocarbons [49]. Huang et al. found that *Methyloversatilis* species were dominant when treating pharmaceuticals and played an important role in ibuprofen removal [50]. Species *SWB02* has the ability to utilize volatile fatty acids from the anaerobic chamber and to enhance the electron transfer between the substance and microbes [51]. Although *SM1A02* species have been studied, many researchers found electrotrophic bacteria which enhanced the electron transfer and correlated with many pharmaceuticals [57,64,65]. Furthermore, species *Emticicia* and *Methylophilus* showed good performance when treating pharmaceuticals [31,58]. Moreover, the abundance of *Acidovorax* was higher in the applied voltage reactors; an electroactive bacterium that is frequently detected in BES [24] and has also been found to be closely related to the removal of pharmaceuticals [35]. *Sporomusa* is a classical type of electroactive bacteria with the ability to develop nanowires for the conduction of electrons [61]. It was found that *Terrimicrobium* was enriched in Rca.An.C higher than in the other reactors, following the same pattern as prior research [63] applied to the bioelectrochemical system for treating groundwater sediment. In our study, the above-mentioned bacteria were capable of degrading pharmaceuticals and were enriched in reactors under electrical stimulation.

As depicted in Figure 2, compared to Rop, the elimination of ibuprofen from Rac and diclofenac from Rca was aided by electric stimulation. The anodic and cathodic chambers were found to have distinct bacteria enriched in them. To further identify the functional microorganisms of different pollutants, the genera in the dominant zones and their proposed functions are listed in Table 4.

For example, *Sporomusa* was enriched in the anaerobic anodic chamber of Rac. In a previous study [61,62], *Sporomusa* was described as a kind of acetogenic microorganism, found to be capable of generating and conducting electrons to the cell. In addition, *SM1A02* is an electrotrophic bacteria and is abundant in the aerobic cathodic chamber of Rac, which was proved to enhance electron transfer and is closely correlated with pharmaceutical removal [56,57,58]. In previous research, Salgado et al. found that electron transfer played important role in enzyme metabolism when treating ibuprofen [66]. Therefore, it was assumed that the enrichment of *Sporomusa* and *SM1A02* enhanced the electron transfer, shortening the bacterial acclimatization time by enhancing electron transfer in the biofilm of the anodic and cathodic chambers of Rac to remove ibuprofen.

*Terrimicrobium* was more abundant in the anaerobic cathodic chamber of Rca than in the anaerobic zone of Rop. In a prior investigation, it was shown capable of degrading polychlorinated biphenyls with a chloro-structure [63]. Additionally, *Methylophilus* was enriched in the aerobic anodic chamber of Rac, and is also a dechlorinating genus [54]. After the favorable impact of electric stimulation, it was found that *Terrimicrobium* and *Methylophilus* were mostly responsible for the elimination of diclofenac.

Furthermore, *Acidovorax* was found in abundance in the anaerobic anodic and cathodic chambers of Rac and Rca. In prior work [35], *Acidovorax* was enriched in the bioelectrochemical system when treating ibuprofen, diclofenac, and carbamazepine. Additionally, *Hydrogenophaga*, *Methyloversatilis*, *SWB02*, and *Emticicia* were all enriched in the chambers of the applied voltage reactors. The above-mentioned bacteria directly or indirectly participate in the removal of pharmaceuticals (by enhancing electron transfer). Therefore, the removal performances of ibuprofen and diclofenac were significantly improved in the AO-UBERs.

## 5. Conclusions

In this study, AO-UBERs achieved complete removal of diclofenac and brought the removal rate of ibuprofen to stability faster than that of the control group under intermittent voltage. The optimal HRT conditions can exert positive electrical stimulation effects on pharmaceuticals removal. Aerobic anodic/cathodic chambers might be reliable for enhancing the treatment efficiency of ibuprofen and diclofenac. Thus, AO-UBERs may offer new ideas for the treatment of PPCPs in agriculture, medical wastewater, and WWTPs. Furthermore, different biodegradation-related species were enriched in the two electrode conversion types, which may represent one of the critical factors for the enhanced performance of different pharmaceuticals. This study provides a broader foundation for promoting the practical application of BESs and a strategy for the efficient removal of PPCPs in sewage. However, the energy consumption of the reactor and the effect of electrical stimulation on the degradation of metabolites from pollutants remain poorly understood and need to be further explored.

## Figures and Tables

**Table 1:** Operational conditions of the reactors.

Reactor	Anaerobic Zone	Aerobic Zone	Voltage Applied Modes	Notes
Rac	Anodic chamber	Cathodic chamber	0, 0.3, 0.6, 0.9, 1.2 V	R1, R2
Rca	Cathodic chamber	Anodic chamber	0, 0.3, 0.6, 0.9, 1.2 V	R3, R4
Rop	Anaerobic zone	Aerobic zone	No applied voltage	R5, R6

**Table 2:** Q-PCR quantification of total microbial community in the biofilm samples.

Sample ID	Sampling Site	log_10_ (Copies/g CFN or g CFB)
Rac.Ae.C	Rac_ aerobic cathode	10.4
Rca.Ae.A	Rca_ aerobic anode	10.3
Rop.Ae.O	Rop_ aerobic open circuit	10.2
Rac.An.A	Rac_ anaerobic anode	10.1
Rca.An.C	Rca_ anaerobic cathode	10.2
Rop.An.O	Rop_ anaerobic open circuit	10.2

**Table 3:** Species diversity and abundance index of eight samples.

Sample ID	Sampling Site	OTUs	Evenness
S.Ae	Sludge_aerobic	271	0.66
Rac.Ae.C	Rac_aerobic cathode	274	0.94
Rca.Ae.A	Rca_aerobic anode	289	0.95
Rop.Ae.O	Rop_aerobic open circuit	275	0.92
S.An	Sludge_anaerobic	235	0.70
Rac.An.A	Rac_anaerobic anode	312	0.89
Rca.An.C	Rca_anaerobic cathode	340	0.97
Rop.An.O	Rop_anaerobic open circuit	295	0.86

OTUs; a higher number represents more diversity. Evenness; a higher number represents more evenness.

**Table 4:** The genera in the dominant zones and their proposed function.

Dominant Zone	Genera	MAIN Function	Highlights	References
Aerobic	1. *Hydrogenophaga*	1. Potential for diclofenac and ibuprofen removal. 2. Enhancing dehydrogenase activity and secretion of EPS. 3. Found in electroactive biofilm and uses hydrogen as an electron.	Enriched in the anodic and cathodic chambers	[46,47,48]
	2. *Methyloversatilis*	1. Essential for the oxidation of aromatic hydrocarbons. 2. Has the ability to degrade pharmaceuticals.		[49,50]
	3. *SWB02*	1. Utilizing volatile fatty acids from the anaerobic chamber. 2. May enhance electron transfer.		[51,52]
	4. *Emticicia*	Correlated with pharmaceutical removal		[53]
	5. *Methylophilus*	1. Pharmaceutical biodegradation. 2. Has the ability of dechlorination.	Enriched in the anodic chamber	[54,55]
	6. *SM1A02*	1. Electrotrophic bacteria and enhanced electron transfer. 2. Anammox genus and may be correlated with pharmaceutical removal.	Enriched in the cathodic chamber	[56,57,58]
Anaerobic	1. *Acidovorax*	1. Could grow using inorganics as electron donors. 2. Closely related to pharmaceutical removal.	Enriched in the anodic and cathodic chamber	[35,59,60]
	2. *Sporomusa*	Generating and conducting electrons.	Enriched in the anodic chamber	[61,62]
	3. *Terrimicrobium*	Degrading substances containing -Cl.	Enriched in the cathodic chamber	[63]

## Data Availability

The datasets supporting the conclusions of this article are included within the article and its additional files. Supplementary data for this work can be found in e-version of this paper online. We will be depositing the sequences in BioProject # PRJNA813363 in the NCBI Sequence Read Archive database. The query link as https://www.ncbi.nlm.nih.gov/sra/?term=PRJNA813363%20 (accessed on 7 March 2022).

## Supplementary Materials

The following supporting information can be downloaded at: https://www.mdpi.com/article/10.3390/ijerph192215364/s1, Figure S1: Real photos of up-flow anaerobic-aerobic-coupled bioelectrochemical reactors. (Rac: anaerobic anodic and aerobic cathodic chambers; Rca: anaerobic cathodic and aerobic anodic chambers; Rop: control reactor without applied voltage). Figure S2: The calibration curves of three pharmaceuticals, the regression coefficients were R2 > 0.99. Figure S3: The pH values of the six reactors (Y represented the anaerobic zone while H represented the aerobic zone). Figure S4: The DO concentrations of the six reactors (Y represented the anaerobic zone while H represented the aerobic zone). Figure S5: The total COD efficiencies of Rac, Rca, and Rop. Table S1: The Coverage values of samples.
