# Supplementary material for: Impact of Voltage Application on Degradation of Biorefractory Pharmaceuticals in an Anaerobic–Aerobic Coupled Upflow Bioelectrochemical Reactor
Source: Int J Environ Res Public Health. 2022 Nov 21;19(22):15364. doi: 10.3390/ijerph192215364 (PMC9690855; doi:10.3390/ijerph192215364)
Supplement: Supplementary file 1 [file ijerph-19-15364-s001.zip › ijerph-1999680-supplementary.pdf]

Supporting information

Intermittent electrical stimulation on biodegradation of pharmaceuticals and bacterial community changes

Qiongfang Zhang[1,2,#], Mei Wu[1,2,#], Nuerla Ailijiang[1,2,*], Anwar Mamat[3], Jiali Chang[4], Miao Pu[1,2], Chaoyue Chao[1,2]

[1] Key Laboratory of Oasis Ecology of Education Ministry, College of Ecology and Environment, Xinjiang University, Urumqi 830017, P.R. China

[2] Xinjiang Jinghe Observation and Research Station of Temperate Desert Ecosystem, Ministry of Education, Urumqi 830017, P.R. China

[3] School of Chemical Engineering and Technology, Xinjiang University, Urumqi 830017, P.R. China

[4] Division of Environmental Engineering, School of Chemistry, Resources and Environment, Leshan Normal University, Sichuan 614000, China

*Corresponding author. Tel: +86-10-2111653

Email address: aljnel@xju.edu.cn (Nuerla Ailijiang)

[#]*These authors contributed to the work equally and should be regarded as co-first authors.*

This supplement provides additional information on the analysis methods and supporting results for the main paper, including 5 figures and 1 table.

Real photos of upflow anaerobic-aerobic-coupled bio-electrochemical reactors：

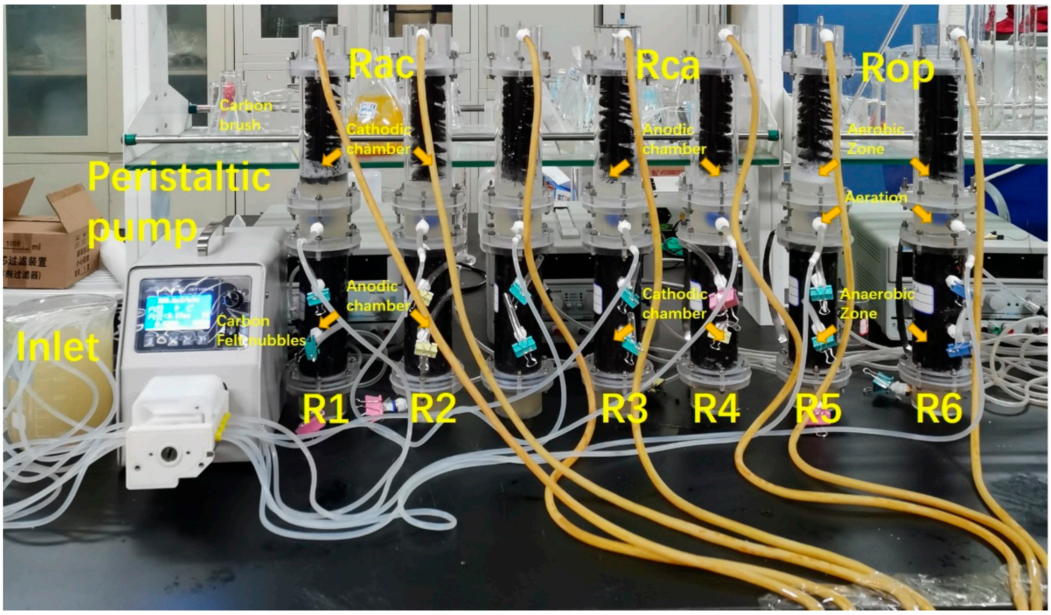

Figure S1. Real photos of up-flow anaerobic-aerobic-coupled bioelectrochemical reactors. (Rac: anaerobic anodic and aerobic cathodic chambers; Rca: anaerobic cathodic and aerobic anodic chambers; Rop: control reactor without applied voltage.)

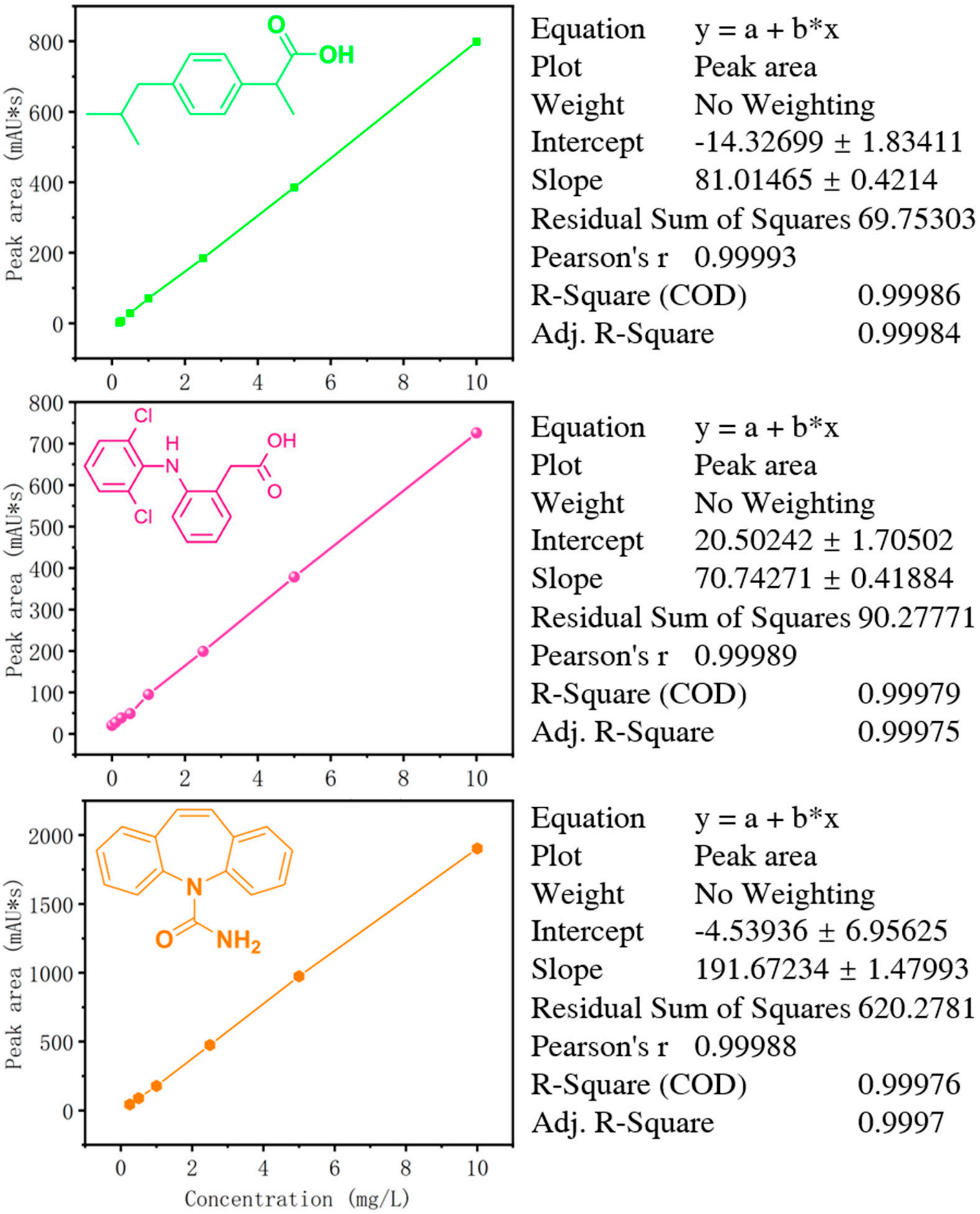

Figure S2. The calibration curves of three pharmaceuticals, the regression coefficients were R^2>0.99.

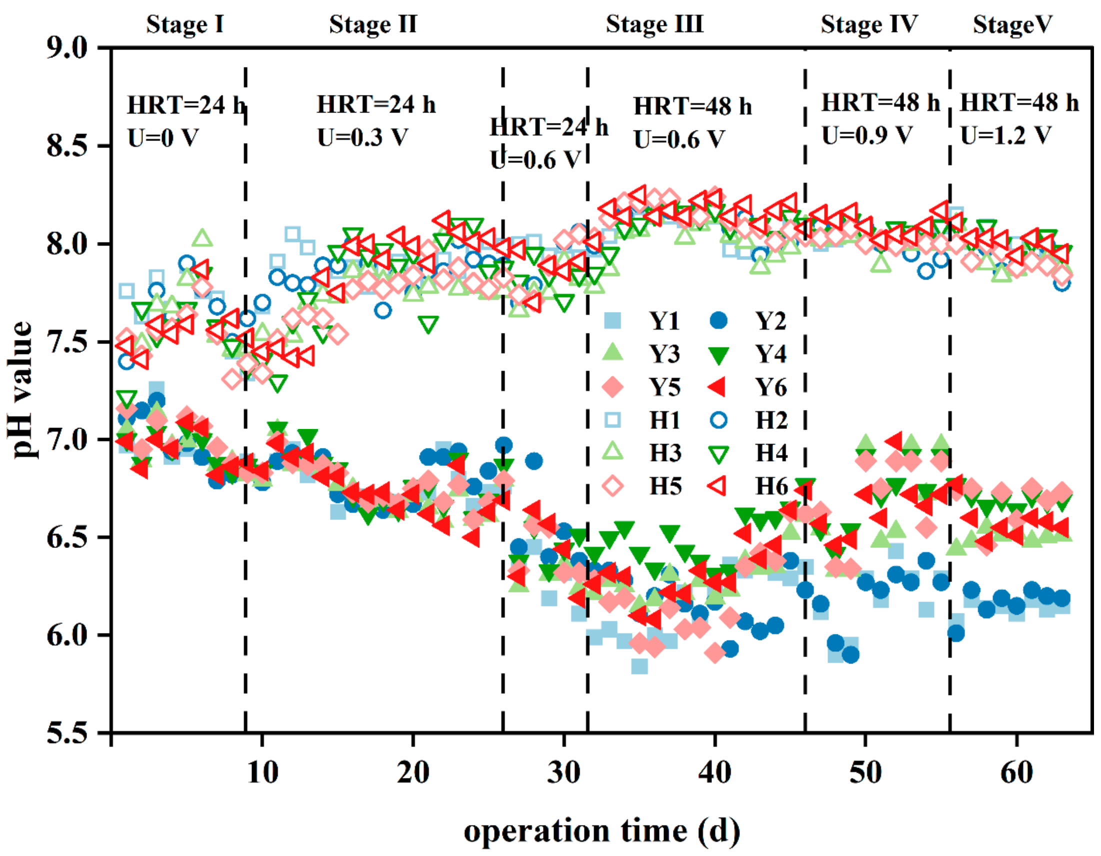

Figure S3. The pH values of the six reactors (Y represented the anaerobic zone while H represented the aerobic zone).

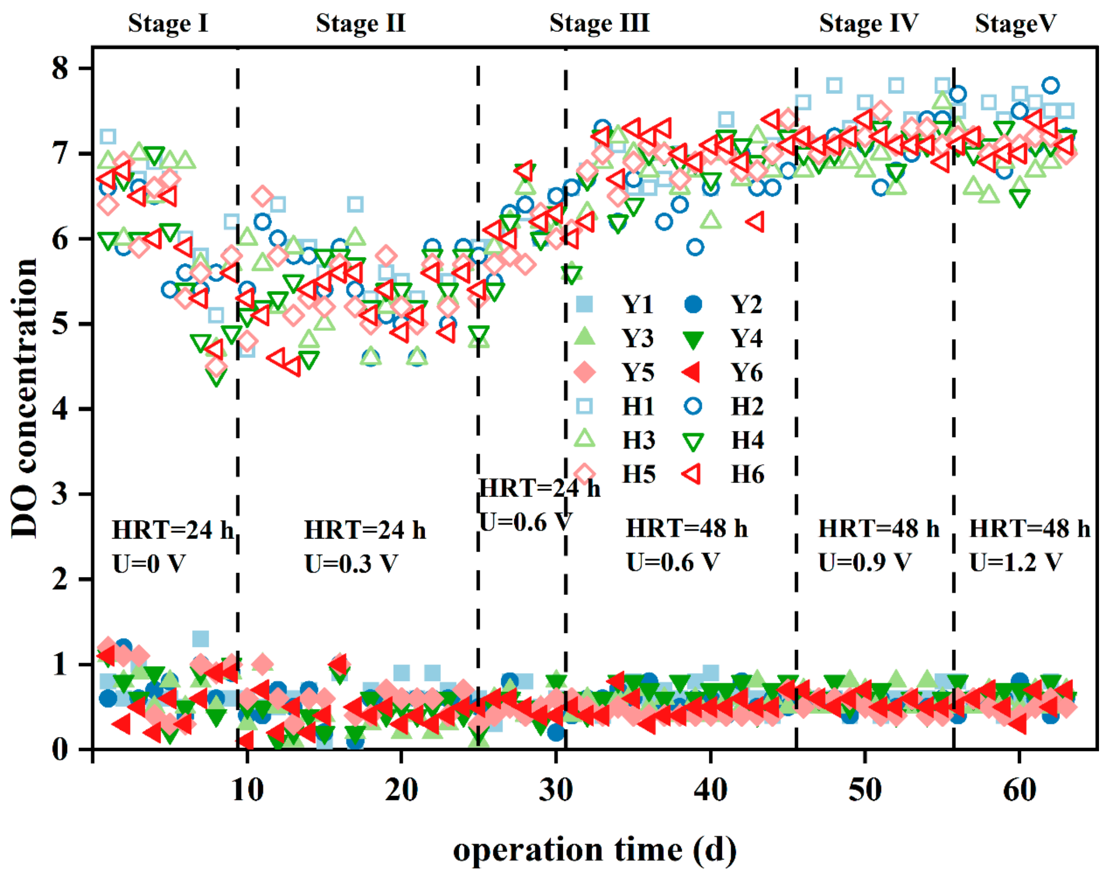

Figure S4. The DO concentrations of the six reactors (Y represented the anaerobic zone while H represented the aerobic zone).

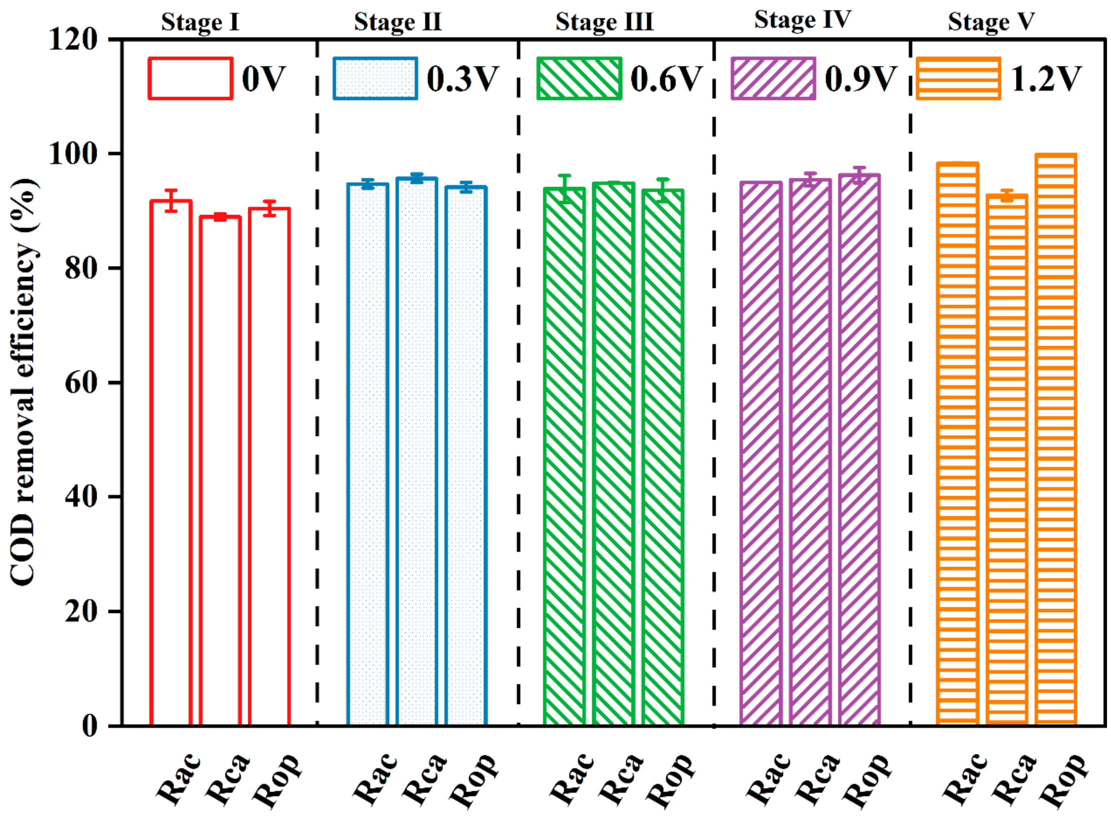

Figure S5. The total COD efficiencies of Rac, Rca, and Rop.

Table S1

Sample ID	Sampling site	Coverage
S.Ae	Sludge_aerobic	0.9994
Rac.Ae.C	Rac_aerobic cathode	0.9990
Rca.Ae.A	Rca_aerobic anode	0.9993
Rop.Ae.O	Rop_aerobic with open circuit	0.9993
S.An	Sludge_anaerobic	0.9989
Rac.An.A	Rac_anaerobic anode	0.9993
Rca.An.C	Rca_aerobic cathode	0.9993
Rop.An.O	Rop_anaerobic with open circuit	0.9993

The Coverage values of samples.
